# MT4‐MMP in tumor‐associated macrophages is linked to hepatocellular carcinoma aggressiveness and recurrence

**DOI:** 10.1002/ctm2.162

**Published:** 2020-08-26

**Authors:** Feng Qi, Jia Li, Mengzhou Guo, Biwei Yang, Jinglin Xia

**Affiliations:** ^1^ Liver Cancer Institute Zhongshan Hospital Fudan University Shanghai P. R. China; ^2^ The First Affiliated Hospital of Wenzhou Medical University Wenzhou P. R. China

Dear Editor,

Hepatocellular carcinoma (HCC) is among the deadliest human cancers, ranking as the fifth most common primary cancer and the third most common cancer‐associated cause of death.^1^ The tumor microenvironment has been proposed in recent years to be closely related to resistance and tumor recurrence.^2^ It includes inflammatory cells, cancer cells, and extracellular matrix (ECM).^3^ Polarization and phenotypic transition of macrophages are the primary features of tumor‐associated chronic inflammation.^4^ Tumor‐associated macrophages (TAMs) show an M2‐like phenotype and are closely involved in tumorigenesis, metastasis, ECM remodeling, and poor prognosis.^5^

MT4‐MMP (also named MMP17), as a member of the MMP family of ECM remodelers, is membrane anchored by a glycosylphosphatidylinositol anchor (GPI)^6^ and directly resolves a specific set of substrates, such as fibrinogen, proTNF, ADAMTS4, and αM integrin.^7^ MT4‐MMP has been reported to be expressed by macrophages and to be associated with inflammation and angiogenesis.^8^ In particular, the MT4‐MMP transcript was recently observed in gastrointestinal adenocarcinomas, prostate cancer, osteosarcomas, leukemias, lung carcinomas, glioblastomas, cervical carcinomas, melanomas, and breast cancer.^9^ However, the role of MT4‐MMP in HCC is still poorly understood.

We explored the MT4‐MMP expression profile in human HCC. Forty‐three and 12 pairs peritumoral and tumor tissues, respectively, were analyzed by qPCR and western blot. Overall survival (OS) was the time from surgery to death or final follow‐up, while recurrence‐free survival (RFS) was defined as the interval between surgery and recurrence. Data revealed that the MT4‐MMP mRNA and protein level was obviously upregulated in peritumoral tissues over tumor tissues (Figure 1A and B). Next, 316 paired peritumoral and tumor tissues were further analyzed for MT4‐MMP expression by immunohistochemistry (Figure 1C). Patient clinical and demographic characteristics are compiled in Table S1. Survival analyses showed that patients in the peritumoral MT4‐MMP‐high group had markedly decreased median OS and RFS (median OS, 58.0 months; RFS, 24.0 months) relative to patients in the peritumoral MT4‐MMP‐low group (median OS, undefined, *P* = .008; RFS, 41.0 months, *P* = .014). In contrast, there was no significant difference between the OS and RFS of the tumoral MT4‐MMP‐high and ‐low groups (median OS, 71 vs 77 months, *P* = .644; RFS, 27 vs 31 months, *P* = .795) (Figure 1D). Multivariate analyses also showed that peritumoral MT4‐MMP was independently associated with OS (HR = 1.957, *P* = .006) and RFS (HR = 1.640, *P* = .015) in HCC patients (Table S2). To assess MT4‐MMP location, we performed colocalization immunofluorescence staining in human HCC tissues, including peritumoral and tumor tissues. MT4‐MMP was expressed in TAMs of peritumoral and tumor tissues, as shown by the colocalization of MT4‐MMP with CD68+CD206+ macrophages (Figure 1E). Notably, MT4‐MMP was not expressed in tumor cells (AFP+), fibroblasts (α‐SMA+), or M1 macrophages (iNOS+) (Figure 1F‐H). Therefore, we clarified the clinical value of peritumoral MT4‐MMP by establishing a prognostic nomogram based upon multivariate Cox analyses. The C‐index was 0.737 (95% CI 0.697‐0.777) for OS prediction and the calibration plot for the probability of OS at 3 and 5 years exhibited optimized agreement between the nomogram‐derived predictions and actual observations (Figure 1I).

**FIGURE 1:**
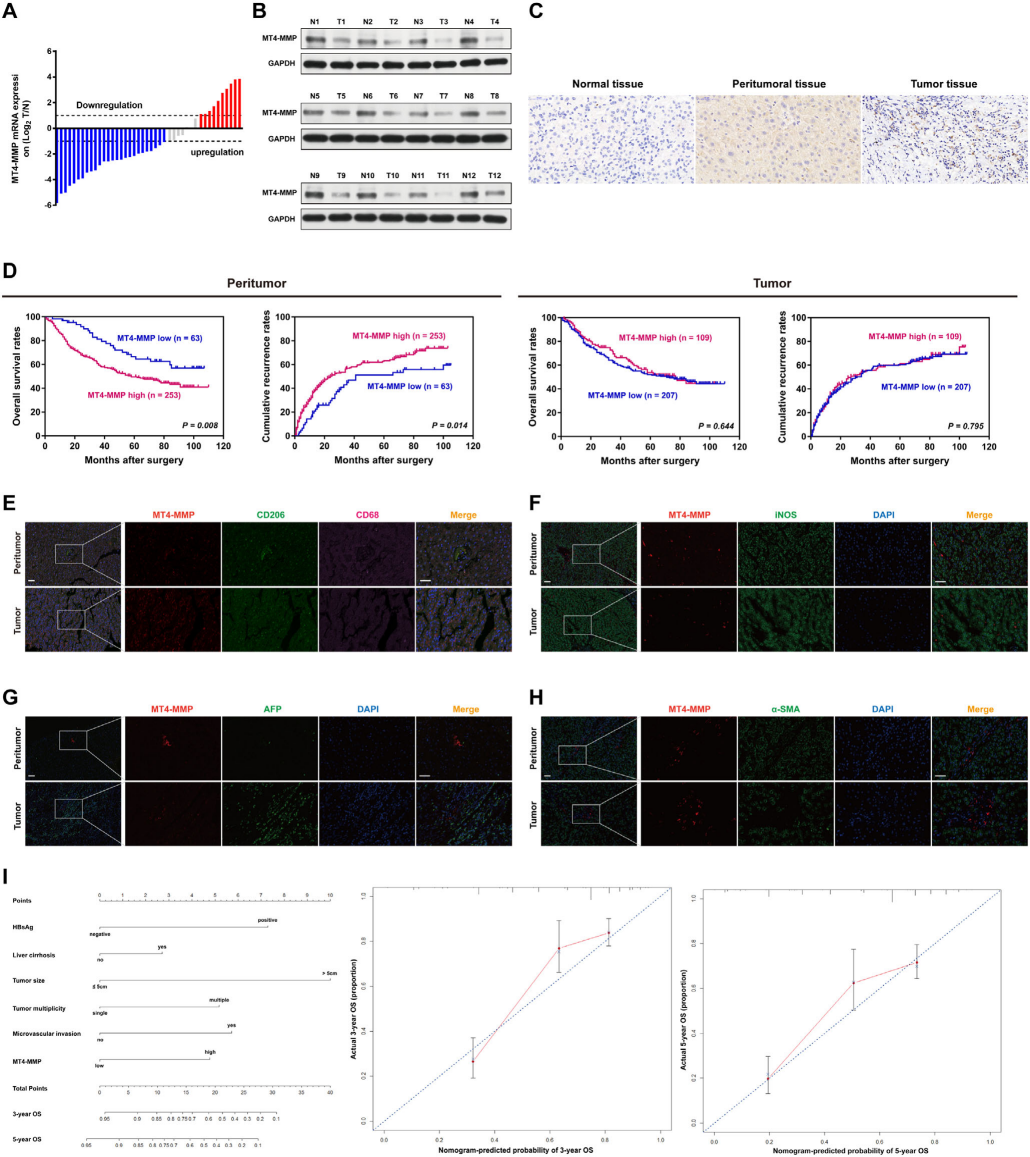
Upregulation of peritumoral MT4‐MMP correlates with poor prognosis in human HCC. A, Waterfall analyses of MT4‐MMP mRNA levels in peritumoral specimens and HCC from 43 patient samples (represented by red, blue, and gray bars). Red and blue bars show a relative MT4‐MMP fold change of more than two for overexpression and underexpression, respectively (T/N, T: tumor, N: nontumor). Gray bars show no difference between peritumoral tissues and HCC (less than twofold change). B, Western blot analysis of MT4‐MMP expression in peritumoral tissues and HCC (n = 12). C, Representative IHC staining pattern of MT4‐MMP in normal liver tissue, peritumoral tissue, and HCC tissue. D, Kaplan‐Meier curves of OS and RFS based on MT4‐MMP expression in peritumoral tissues (left panel) and tumor center areas (right panel) (n = 316). E‐H, Representative fluorescent images showing coimmunostaining of MT4‐MMP in human peritumoral tissues and HCC. MT4‐MMP (red color) was colocalized mainly with CD68+ (pink) and CD206+ macrophages (green) but not with AFP+ HCC (green), α‐SMA+ fibroblasts (green), or iNOS+ macrophages (green). Blue, DAPI. Scale bars = 50 μm. I, Prognostic nomogram based on clinical characteristics for 3‐ and 5‐year OS in HCC patients

In vitro assay, we found that the expression levels of MT4‐MMP in M2 and TAMs were significantly higher than in the M0 and M1 groups (Figure S1). Similarly, MT4‐MMP expression was higher in TAMs than in HCC cells (Figure S2). To assess the role of MT4‐MMP in TAMs for HCC progression, we generated stable MT4‐MMP overexpression and knockdown clones of TAMs (Figure S3). MHCC97H cells were selected and harvested after 48 hours of coculture with TAMs for use in proliferation, apoptosis, and metastasis assays. Compared with the control cells, TAMs with increased MT4‐MMP expression markedly promoted the growth and reduced the apoptosis of HCC cells, whereas TAMs with knockdown of MT4‐MMP expression lowered cell viability and increased cell apoptosis (Figure 2A and B). The stable overexpression of MT4‐MMP in TAMs contributed to the accumulation of cell motility, migration, and invasion in HCC cells, while the suppression of MT4‐MMP expression produced the opposite effect (Figure 2C and D). In view of the critical role of epithelial‐mesenchymal transition (EMT) in tumorigenesis, metastasis, and recurrence, we explored EMT marker expression in control and hepatoma cells cocultured with TAMs with MT4‐MMP overexpression or knockdown. IF staining demonstrated that TAMs overexpressing MT4‐MMP had reduced epithelial E‐cadherin expression and increased mesenchymal N‐cadherin and vimentin expression, whereas knockdown of MT4‐MMP yielded the opposite phenotype (Figure 2E).

FIGURE 2HCC‐conditioned TAMs promote oncogenic behaviors of MHCC97H cells in vitro. MHCC97H cells were coincubated with TAM cells for 48 hours, and then cell experiments were conducted. (A) The CCK‐8 assay was used to examine cell viability. Upper panel: The effect of MT4‐MMP overexpression in TAMs on the cell proliferation ability of MHCC97H cells was tested by CCK‐8 assay. Lower panel: The influence of MT4‐MMP knockdown in TAMs on the cell proliferation ability of MHCC97H cells was tested by CCK‐8 assay. B, MHCC97H cell apoptosis after coculture by means of flow cytometry analysis of annexin‐V/PI staining. Left panel: MHCC97H cells distributed in annexin V scatter plots. The red box represents the late‐apoptotic cells in the upper right quadrant and shows the early‐apoptotic cells in the lower right quadrant. The upper left quadrant shows the necrotic cells, and the lower left quadrant shows the live cells. Right panel: Quantification of cell apoptosis. C, The scratch assay was used to examine cell migration. Left panel: Representative images of the scratch assay showing that TAMs with MT4‐MMP overexpression (MT4‐MMP) promoted the migration of MHCC97H cells in vitro and TAMs with MT4‐MMP knockdown (KD1 and KD2) inhibited the migration of MHCC97H cells in vitro. Right panel: Quantification of the results of the scratch assay, ×100 magnification. (D) Transwell migration and invasion assays showed that TAMs overexpressing MT4‐MMP promoted the migration and invasion of MHCC97H cells. Left panel: The bar graphs represent the number of migrating and invading cells after 48 hours. Right panel: Quantification of the results of transwell assays, × 100 magnification. (E) Immunofluorescence staining of EMT‐related markers showed that TAMs overexpressing MT4‐MMP promoted EMT in MHCC97H cells in vitro. Left panel: Representative images of EMT‐related markers in each group, scale bar = 50 μm. Right panel: Quantification of EMT‐related marker staining in MHCC97H cells. All histogram bars represent mean ± SEM. *P < .05, **P < .01, ***P < .001 versus the control group (Ctrl). Three experimental replicates in all panels

**Figure:**
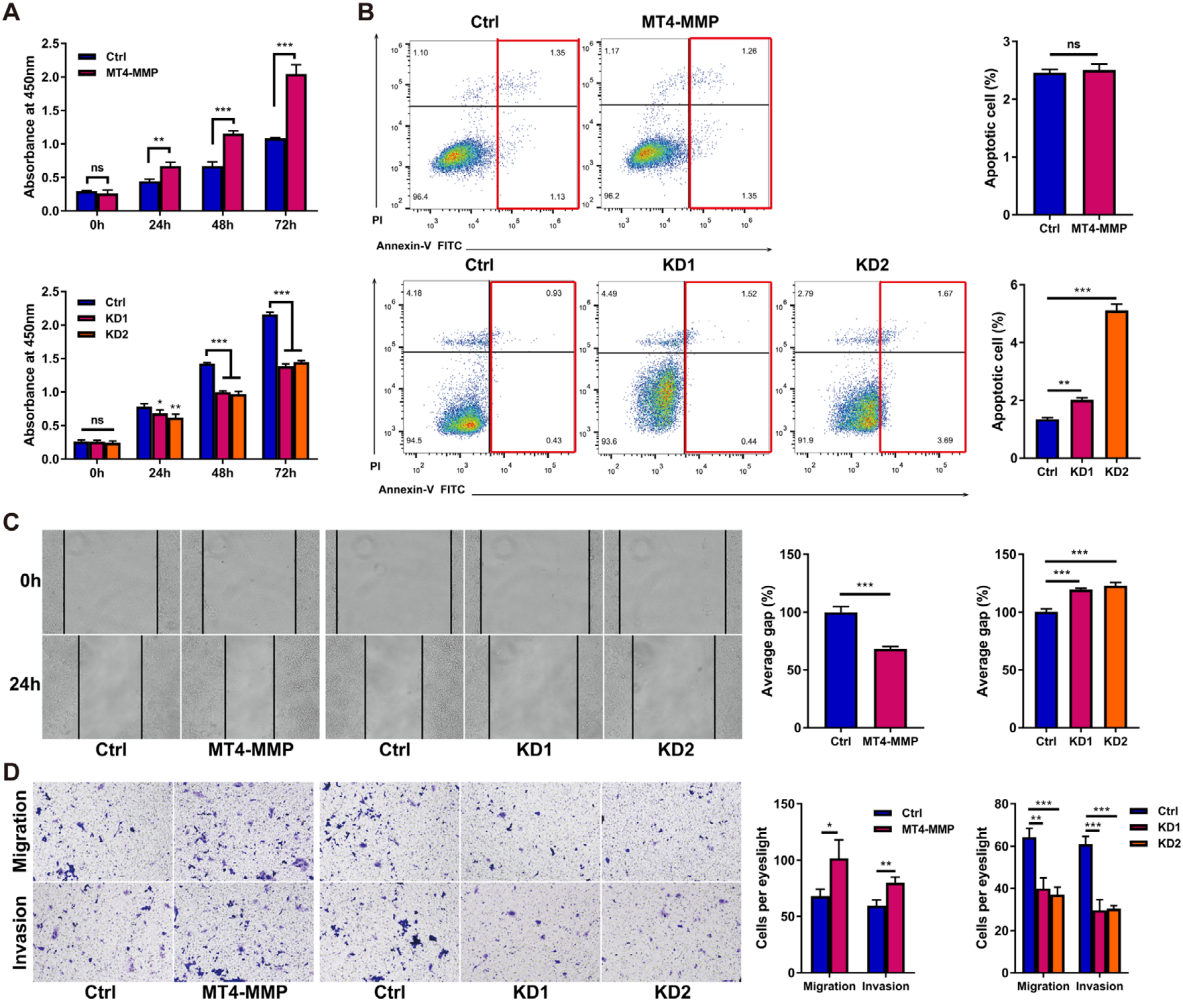

**Figure:**
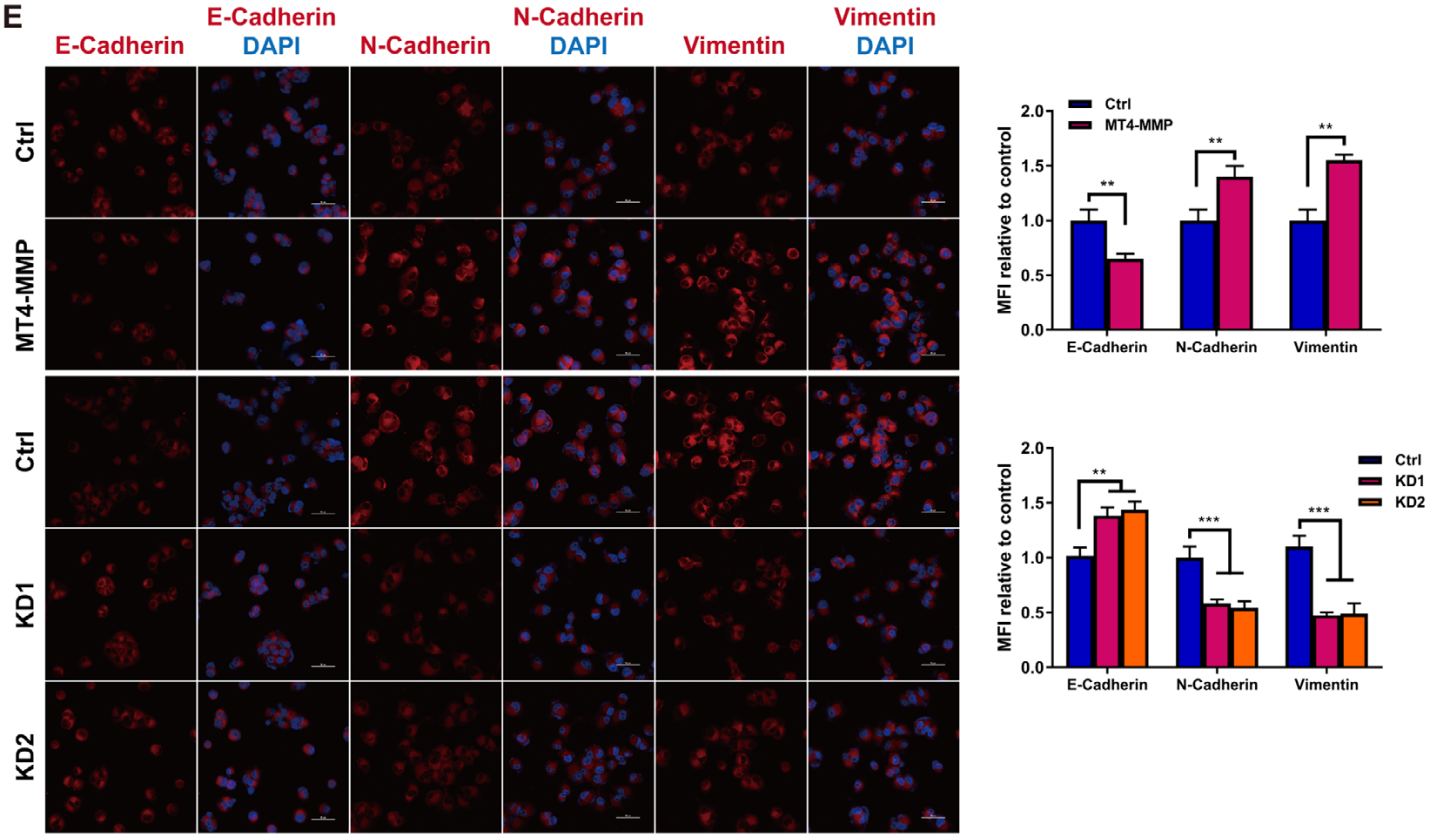

In conclusion, we have identified that high MT4‐MMP expression in peritumoral tissues has a strong relationship with HCC aggressiveness and recurrence and can be seen in TAMs. Additionally, through in vitro experiments, we verified that MT4‐MMP in TAMs promoted HCC proliferation and metastasis. Hence, MT4‐MMP in TAMs may become a potential biomarker for predicting prognosis and may be a promising therapeutic target for HCC.

## CONFLICT OF INTEREST

The authors declare that there is no conflict of interest.

## AUTHOR CONTRIBUTIONS

Feng Qi and Jinglin Xia designed the study and analyzed and wrote the manuscript. Jia Li and Mengzhou Guo conducted the in vitro experiment and data analysis. Feng Qi and Biwei Yang collected the patient samples and follow‐up information and performed the clinical data analysis. Feng Qi, Jia Li, and Biwei Yang performed the bioinformatics analysis. All the authors read and approved the final manuscript.

## ETHICS APPROVAL AND CONSENT TO PARTICIPATE

All protocols with human specimens were applied under the examination and approval of the Ethical Committee of Zhongshan Hospital, Fudan University. Written informed consent was provided by each patient (B2020‐051).

## FUNDING INFORMATION

National Natural Science Foundation of China; Grant Nos. 81772590 and 81572395.

## Supporting information

Supporting Information
