# Supplementary material for: MT4‐MMP in tumor‐associated macrophages is linked to hepatocellular carcinoma aggressiveness and recurrence
Source: Clin Transl Med. 2020 Aug 26;10(4):e162. doi: 10.1002/ctm2.162 (PMC7449244; doi:10.1002/ctm2.162)
Supplement: Supplementary file 1 — Supporting Information [file CTM2-10-e162-s001.docx]

# Supplementary information

**Materials and Methods**

*Real-time quantitative PCR*

Total RNA from HCC tissues and cells were obtained by Trizol (Takara, Japan). Then, Reverse-transcription was performed by PrimeScript RT reagent kit (Takara, Japan). Reactions were measured by using the SYBR® Premix ExTaq™ (Takara, Japan) on an ABI Prism 7500 Sequence Detection system (Applied Biosystems, Foster City, CA, USA). The sequences of primers were listed as follow: MT4-MMP forward 5’-CTGGGAGTGGAGTGGCTAAGCA-3’. Reverse 5’- TTTCATCAGGGCCAGGGTGG-3’; GAPDH forward 5’- CTGGGCTACACTGAGCACC-3’. Reverse 5’-AAGTGGTCGTTGAGGGCAATG -3’. The glyceraldehyde 3-phosphate dehydrogenase (GAPDH) was used as an internal control for normalization by standard 2−ΔΔCT calculation. All experiments were repeated three times.

*Western blot*

Tissues and cells were lysed in the RIPA extraction regent (Pierce Biotechnology, Rockford, IL, USA) for 30 min on ice. After centrifugation at 12000rpm for 10min, the supernatant collected was added with 5 × SDS-PAGE loading buffer (Beyotime, Shanghai, China. Cat No#P0015) and denatured by boiling. Protein samples were loaded per well and electrotransfered to polyvinylidene fluoride membranes. After sealing with 5% non-fat milk for 1h, membranes were incubated with primary antibodies (anti-MT4-MMP, 1:500 dilution, JU33-03, Novus, CO, USA) at 4 °C overnight and then incubated with the corresponding secondary antibodies (1:5000 dilution, Jackson ImmunoResearch Laboratories, West Grove, PA) for 1h at room temperature. The membranes were exposed to image by Tanon-5200 Chemiluminescent Imaging System (Tanon, China). All experiments were repeated three times.

*Immunohistochemistry and tissue microarray analysis*

As described previously, a tissue microarray (TMA) was prepared and IHC analyses were conducted.17 Tissue sections were subjected to antigen repair in a microwave in an EDTA-based antigen repair buffer (pH = 9.0). Sections were incubated with 3% hydrogen peroxide solution at room temperature and in the dark for 20 min. Then, the tissue was covered with 5% BSA with MMP17 (MT4-MMP) antibody (1:100, JU33-03, Novus, CO, USA) and incubated at 4°C overnight. After that, the sections were covered with the second antibody (HRP-labeled) corresponding to the primary antibody (1:100, Abcam, Cambridge) and incubated for 50 min. Then DAB was applied, and Harris’s hematoxylin was used for nuclear counterstaining.

All tissue areas were evaluated by the same criteria, including percentage of cell positivity and staining intensity. Positive cells needed to be differentiated from nonspecific staining. Staining intensity was scored as follows: 0 for no staining, 1 for light yellow, 2 for dark yellow, and 3 for brown. The cell-positive ratio was used for scoring: 0 ~ 5% for score 0, 6 ~ 25% for 1, 26 ~ 50% for 2, 51 ~ 75% for 3, and >75% for 4. The comprehensive positive score (CPS) was obtained by multiplying together percept positivity and staining intensity scores: 0 was negative (-), 1 ~ 4 was weakly positive (+), 5 ~ 8 was moderately positive (++), and 9 ~ 12 was strongly positive (+++). Based on the final score, a high MT4-MMP expression level was defined as CPS > 4 and low as CPS ≤ 4.

*Immunofluorescence*

Tissue sections or cell slides were added to EDTA antigen repair buffer (pH 8.0) and then incubated with MMP17 (MT4-MMP) antibody (1:100, JU33-03, Novus, CO, USA), CD206 (1:500, ab64693, Abcam, USA), CD68 (1:100, ab213363, Abcam, USA), α-SMA (1:100, ab32575, Abcam, USA), AFP (1:100, ab133617, Abcam, USA) or iNOS (1:100, ab213987, Abcam, USA) overnight at 4°C. After that, the coverslips were covered with the second antibody of the corresponding first antibody and incubated for 50 min. DAPI dye was dripped into the cover glass ring and incubated for 10 min protected from light, after which a fluorescence microscope (Olympus) was used for sample analysis.

*Cell lines and culture*

THP-1 and L02 cells were from the cell bank of the Chinese Academy of Sciences (Shanghai, China). MHCC97H cells were established at the Liver Cancer Institute of Zhongshan Hospital, Fudan University. Hepatoma cells and L02 cells were maintained in DMEM (Invitrogen), and THP-1 cells were cultured in RPMI-1640 (GE Healthcare). All media contained 10% FBS (GIBCO). M0, M1, M2 and TAM cells were differentiated as previously described [1] and cultured in RPMI-1640 containing 10% FBS.

*Construction of Lentivirus-mediated MT4-MMP knockdown and overexpression clones*

MT4-MMP overexpression and knockdown stable clones were established via lentiviral-mediated expression and RNA interference, respectively. A panel of lentiviral particles with MT4-MMP target knockdown sequences and MT4-MMP cDNA fragment were constructed as previously described [2] and purchased from Heyuan Biotechnology Company (Shanghai, China). The 6-well plates were used to seed TAMs at a density of 5 × 105 cells per well in RPMI-1640 medium supplemented with 10% fetal bovine serum and then transfected at a MOI of 50 and cell culture supernatants were replaced with fresh medium after a 12h incubation with lentivirus. Then, the efficiency of overexpression or knockdown was examined by western blot analysis.

*Flow cytometric analysis*

Cells were collected and washed three times by PBS. Then, after [centrifugation](javascript:;), binding buffer (BD Biosciences), propidium iodide (PI, Invitrogen) and FITC-conjugated Annexin V (BioVision) were used to stain the cells for 30 minutes at room temperature. Apoptosis percentage was determined using BD Accuri C6 flow cytometer and FACSDiva software (BD Biosciences). The experiments were repeated at least three times.

*Cell proliferation, migration and invasion assays*

After co-culturing, MHCC97H cells were planted in 96-well plates at 5000 cells/well and observed the proliferation at 12h, 24, 48h and 72h. Cell viability was detected by using the Cell Counting Kit-8 (Dojindo, Japan) according to the manufacturer’s protocol. The capacity of cell migration and growth was detected by scratch assay and transwell migration and invasion assay. MHCC97H cells in 6-well plates were scratched into the cell monolayer with 200ul tips and a wound was generated. After incubating with medium with FBS for 24h, cells were photographed by a microscope. Cell suspension was prepared. 10^4^ cells were seeded into the top chamber of each insert in serum-free medium and 20% serum-containing medium was used in the lower chamber for cell migration assay. For cell invasion assay, 100μl of Matrigel (diluted at 1:5 with DMEM, BD biosciences) was inserted in the top chamber and solidified at 37°C for 4h. After culturing for 24h, the top chamber was washed by PBS and stained by the crystal violet with 4% paraformaldehyde for 15min. The image was captured under a light microscope. All experiments were carried out for three times.

*Statistical analysis*

Continuous and categorical variables were compared via t-tests and chi-squared tests. ANOVAs and Tukey's post hoc tests were used for multi-group comparisons. Continuous data are means ± SE (minimum, maximum), the categorical variables as a percentage. Prognostic factors were evaluated by Cox proportional hazard regression, and HRs with their 95% confidence intervals were calculated. Survival analysis was generated by the Kaplan-Meier method (log-rank tests). Statistical analyses were done with SPSS v22.0 (IBM SPSS Statistics, IL, US). P<0.05 was the significance threshold.

# Reference

1. Li L, Sun P, Zhang C, Li Z, Cui K, Zhou W. MiR-98 modulates macrophage polarization and suppresses the effects of tumor-associated macrophages on promoting invasion and epithelial-mesenchymal transition of hepatocellular carcinoma. Cancer Cell Int. 2018;18: 95.
2. Huang CH, Yang WH, Chang SY, et al. Regulation of membrane-type 4 matrix metalloproteinase by SLUG contributes to hypoxia-mediated metastasis. Neoplasia. 2009;11(12):1371‐1382.

# Figure S1.


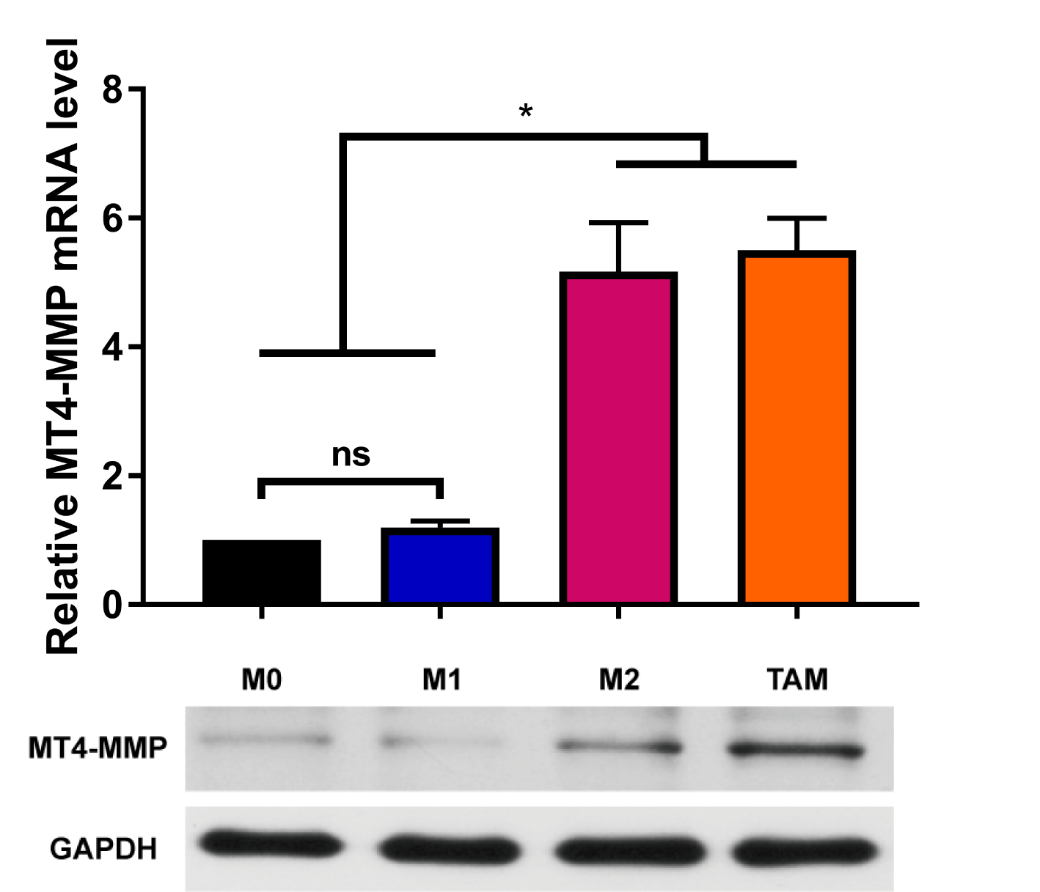


**Figure 1S.** **The protein and mRNA expression of MT4-MMP was significantly upregulated in HCC-conditioned TAMs compared with M0 and M1 cells.**

# Figure S2.


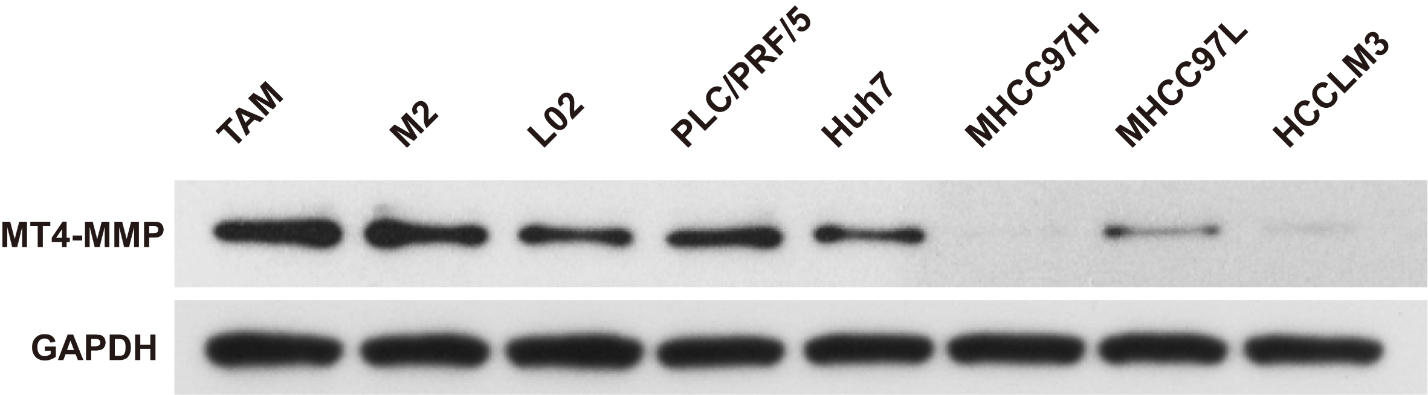


**Figure 2S. The MT4-MMP protein was significantly upregulated in HCC-conditioned TAMs compared with HCC cells.**

# Figure S3.


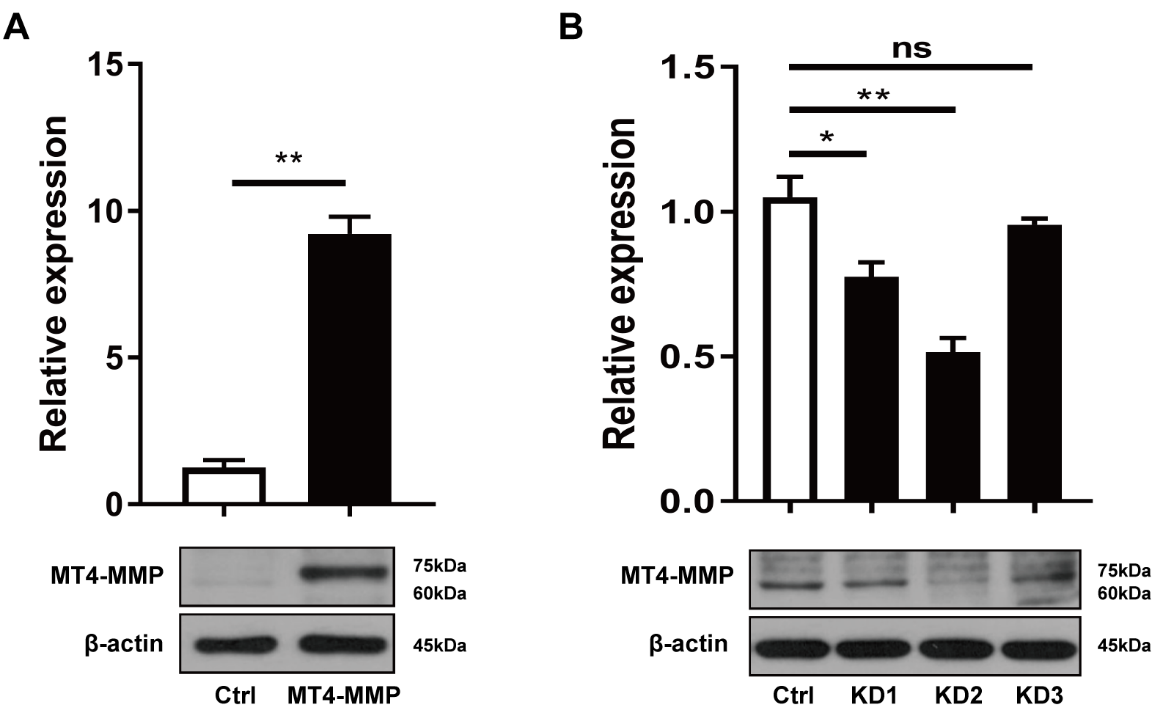


**Figure 3S. Efficiency of MT4-MMP overexpression and knockdown was examined by western blotting.** (A) The protein level revealed the expression of MT4-MMP in overexpression and the control cells. (B) The protein level revealed the efficient knockdown of MT4-MMP expression in KD1 and KD2 cells. β-actin is set as internal control. Quantification of western blotting data was from three separate experiments. *P<0.05, ***P* < 0.01.

# Supplementary Table

Table 1S. Correlations between MT4-MMP expression and clinicopathologic characteristics in HCC patients (n = 316)

| Characteristics | Peritumor | | | Tumor | | |
| --- | --- | --- | --- | --- | --- | --- |
|  | Low | High | *P** | Low | High | *P** |
| **Age, years** |  |  |  |  |  |  |
| ≤50 | 22 | 91 | 0.877 | 84 | 29 | 0.014 |
| >50 | 41 | 162 |  | 123 | 80 |  |
| **Gender** |  |  |  |  |  |  |
| Female | 18 | 26 | <0.001 | 33 | 11 | 0.153 |
| Male | 45 | 227 |  | 174 | 98 |  |
| **HBsAg** |  |  |  |  |  |  |
| Negative | 8 | 47 | 0.308 | 32 | 23 | 0.429 |
| Positive | 53 | 203 |  | 172 | 84 |  |
| Unknown | 2 | 3 |  | 3 | 2 |  |
| **Cirrhosis** |  |  |  |  |  |  |
| No | 25 | 97 | 0.845 | 76 | 46 | 0.341 |
| Yes | 38 | 156 |  | 131 | 63 |  |
| **AFP (ng/ml)** |  |  |  |  |  |  |
| ≤20 | 29 | 108 | 0.818 | 86 | 51 | 0.622 |
| >20 | 34 | 141 |  | 119 | 56 |  |
| Unknown | 0 | 4 |  | 2 | 2 |  |
| **ALT (U/L)** |  |  |  |  |  |  |
| ≤40 | 41 | 158 | 0.420 | 134 | 65 | 0.491 |
| >40 | 21 | 81 |  | 65 | 37 |  |
| Unknown | 1 | 14 |  | 8 | 7 |  |
| **Tumor size (cm)** |  |  |  |  |  |  |
| ≤5 | 44 | 140 | 0.037 | 120 | 64 | 0.898 |
| >5 | 19 | 113 |  | 87 | 45 |  |
| **Tumor encapsulation** |  |  |  |  |  |  |
| None | 27 | 138 | 0.251 | 100 | 65 | 0.157 |
| Complete | 35 | 112 |  | 104 | 43 |  |
| Unknown | 1 | 3 |  | 3 | 1 |  |
| **Tumor multiplicity** |  |  |  |  |  |  |
| Single | 51 | 208 | 0.816 | 168 | 91 | 0.609 |
| Multiple | 12 | 45 |  | 39 | 18 |  |
| **Microvascular invasion** |  |  |  |  |  |  |
| No | 59 | 228 | 0.385 | 184 | 103 | 0.101 |
| Yes | 4 | 25 |  | 23 | 6 |  |
| **Tumor** **differentiation** |  |  |  |  |  |  |
| I-II | 43 | 155 | 0.369 | 128 | 70 | 0.753 |
| III-IV | 20 | 93 |  | 75 | 38 |  |
| Unknown | 0 | 5 |  | 4 | 1 |  |
| **BCLC stage** |  |  |  |  |  |  |
| 0+A | 34 | 143 | 0.715 | 118 | 59 | 0.624 |
| B+C | 29 | 110 |  | 89 | 50 |  |

**Abbreviations:** HCC, hepatocellular carcinoma; HBsAg, hepatitis B surface antigen; AFP, α-fetoprotein; ALT, alanine transaminase; BCLC, Barcelona Clinic Liver Cancer. *P* < 0.05 was considered statistically significant, Pearson χ2 tests.

Table 2S. Univariate and multivariate analyses of prognostic factors associated with OS and recurrence in HCC patients (n = 316).

| Variables | OS | | | | RFS | | | |
| --- | --- | --- | --- | --- | --- | --- | --- | --- |
|  | Univariate | Multivariate | | | Univariate | Multivariate | | |
|  | *P** | HR | 95%CI | *P** | *P** | HR | 95%CI | *P** |
| Age, years (>50 vs. ≤50) | 0.869 | 1.149 | 0.803-1.646 | 0.447 | 0.899 | 1.255 | 0.903-1.745 | 0.177 |
| Gender (female vs. male) | 0.485 | 1.112 | 0.658-1.881 | 0.692 | 0.084 | 0.790 | 0.484-1.291 | 0.348 |
| HBsAg (positive vs. negative) | **0.014** | 3.099 | 1.802-5.331 | **<0.001** | **0.005** | 2.682 | 1.669-4.310 | **<0.001** |
| Liver cirrhosis (yes vs. no) | 0.093 | 1.495 | 1.042-2.146 | **0.029** | 0.059 | 1.316 | 0.955-1.813 | 0.093 |
| Serum AFP, ng/ml (>20 vs. ≤20) | **0.004** | 1.294 | 0.897-1.866 | 0.168 | 0.098 | 1.117 | 0.802-1.556 | 0.512 |
| Serum ALT, U/L (>40 vs. ≤40) | 0.208 | 1.016 | 0.717-1.440 | 0.928 | 0.168 | 1.102 | 0.799-1.519 | 0.553 |
| Tumor size (cm) (>5 vs. ≤5) | **<0.001** | 3.551 | 2.486-5.073 | **<0.001** | **0.002** | 1.617 | 1.160-2.254 | **0.005** |
| Tumor encapsulation (none vs. complete) | 0.190 | 1.223 | 0.863-1.734 | 0.257 | **0.026** | 1.507 | 1.096-2.070 | **0.012** |
| Tumor multiplicity (multiple vs. single) | **0.006** | 1.829 | 1.236-2.705 | **0.003** | **<0.001** | 2.165 | 1.516-3.091 | **<0.001** |
| Tumor differentiation (III-IV vs. I-II) | 0.102 | 1.141 | 0.805-1.618 | 0.459 | 0.567 | 1.004 | 0.727-1.388 | 0.978 |
| Microvascular invasion (yes vs. no) | **<0.001** | 1.688 | 1.009-2.823 | **0.046** | **<0.001** | 1.605 | 0.943-2.733 | 0.081 |
| Peritumor MT4-MMP level (high vs. low) | **0.009** | 1.957 | 1.217-3.147 | **0.006** | **0.016** | 1.640 | 1.099-2.447 | **0.015** |
| Tumor MT4-MMP level (high vs. low) | 0.646 | 0.921 | 0.646-1.313 | 0.650 | 0.797 | 1.072 | 0.776-1.481 | 0.675 |

**Abbreviations:** HCC, hepatocellular carcinoma; OS, overall survival; RFS, recurrence-free survival; HBsAg, hepatitis B surface antigen; AFP, α-fetoprotein; ALT, alanine transaminase; HR, hazard ratio; 95%CI, 95% confidential interval. *P* value* < 0.05 was considered statistically significant
